# Mechanistic View on the Effects of SGLT2 Inhibitors on Lipid Metabolism in Diabetic Milieu

**DOI:** 10.3390/jcm11216544

**Published:** 2022-11-04

**Authors:** Habib Yaribeygi, Mina Maleki, Željko Reiner, Tannaz Jamialahmadi, Amirhossein Sahebkar

**Affiliations:** 1 Research Center of Physiology, Semnan University of Medical Sciences, Semnan, Iran; 2 Urology and Nephrology Research Center, Shahid Beheshti University of Medical Sciences, Tehran, Iran; 3 Department of Internal Medicine, University Hospital Center Zagreb, 1000 Zagreb, Croatia; 4 Applied Biomedical Research Center, Mashhad University of Medical Sciences, Mashhad, Iran; 5 Biotechnology Research Center, Pharmaceutical Technology Institute, Mashhad University of Medical Sciences, Mashhad, Iran; 6 Department of Biotechnology, School of Pharmacy, Mashhad University of Medical Sciences, Mashhad, Iran

**Keywords:** diabetes mellitus, sodium-glucose cotransporter-2 inhibitors, lipids, cholesterol, lipogenesis, lipolysis, β-oxidation, oxidative stress

## Abstract

Chronic hyperglycemia induces pathophysiologic pathways with negative effects on the metabolism of most substrates as well as lipids and lipoproteins, and thereby induces dyslipidemia. Thus, the diabetic milieu is commonly accompanied by different levels of atherogenic dyslipidemia, which is *per se* a major risk factor for subsequent complications such as atherosclerosis, coronary heart disease, acute myocardial infarction, ischemic stroke, and nephropathy. Therefore, readjusting lipid metabolism in the diabetic milieu is a major goal for preventing dyslipidemia-induced complications. Sodium-glucose cotransporter-2 (SGLT2) inhibitors are a class of relatively newly introduced antidiabetes drugs (including empagliflozin, canagliflozin, dapagliflozin, etc.) with potent hypoglycemic effects and can reduce blood glucose by inducing glycosuria. However, recent evidence suggests that they could also provide extra-glycemic benefits in lipid metabolism. It seems that they can increase fat burning and lipolysis, normalizing the lipid metabolism and preventing or improving dyslipidemia. Nevertheless, the exact mechanisms involved in this process are not well-understood. In this review, we tried to explain how these drugs could regulate lipid homeostasis and we presented the possible involved cellular pathways supported by clinical evidence.

## 1. Introduction

The global incidence of diabetes mellitus (DM) is growing rapidly [1]. This chronic disease has negative effects on most cellular metabolic events and induces a series of pathophysiological pathways causing complications in diabetes [2]. It changes the normal metabolism of different substrates, among them lipids (e.g., triglycerides (TGs) and free fatty acids (FFAs)) [3]. Lipids are important biological molecules that have important structural and physiological roles in body homeostasis. They are considered as the main metabolic substrates that store and produce high amounts of required energy for cellular activities [4,5]. However, their normal metabolism is changed in the diabetic milieu and diabetes could induce and promote dyslipidemia-dependent complications (e.g., cardiovascular and renal diseases) [2,5,6]. Therefore, normalizing lipid metabolism in the diabetic milieu is important to reduce the levels of harmful by-products and cellular damage and to prevent dyslipidemia-induced complications of diabetes [2,7].

Sodium-glucose cotransporter-2 inhibitors (SGLT2is) are a class of relatively newly developed antidiabetes drugs that reduce serum glucose concentration by inducing glycosuria [8]. They are effective glucose-lowering drugs that reduce serum glucose almost to the physiological levels [8]. Emerging evidence suggests that they may also provide extra glycemic-lowering benefits [9,10,11,12,13,14,15], and that they may be able to modulate lipid metabolism [16]. If this is true, SGLT2is can provide dual benefits on glucose and lipid homeostasis, which would position them as effective antidiabetes drugs that normalize blood glucose but also as drugs, which might prevent dyslipidemia-associated complications of diabetes. However, the exact mechanism by which these drugs achieve beneficial effects and the involved mechanisms are not well-understood. Therefore, in this review, we discuss the possible effects of SGLT2 inhibitors on lipid metabolism in the diabetic milieu.

## 2. Sodium-Glucose Cotransporter-2 Inhibitors

**Figure 1:**
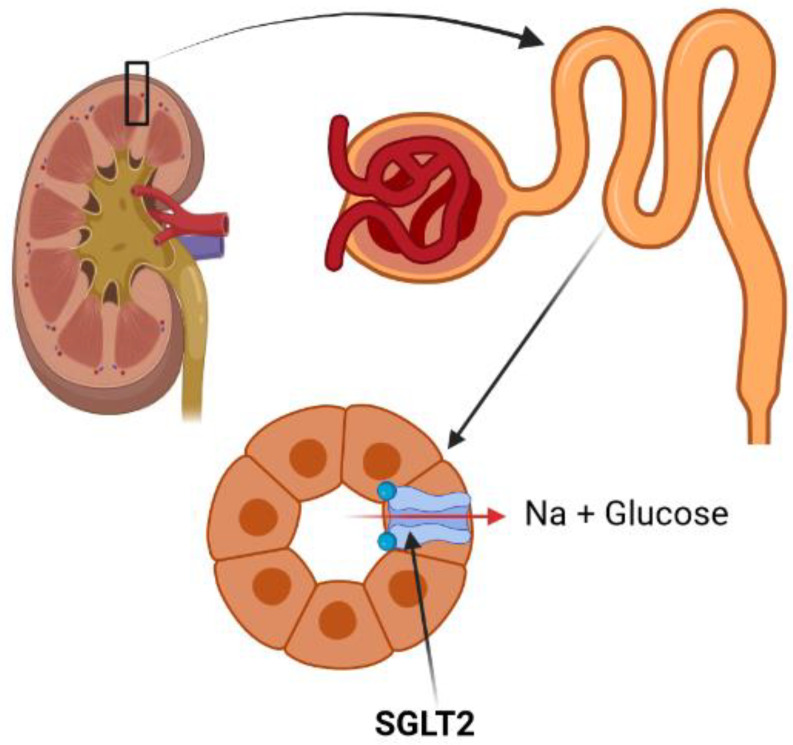
SGLT2 is responsible for glucose and sodium reabsorption in the renal proximal tubules. SGLT2 inhibitors inhibit this process and induce urinary glucose (and sodium) excretion.

SGLT2 inhibitors are a class of relatively newly introduced antidiabetic drugs that decrease blood glucose by the inhibition of renal glucose reabsorption and by inducing urinary glucose excretion (Figure 1) [17,18]. Sodium-glucose co-transporters are two forms of active cotransporters (types 1 and 2) with specific expression in the intestine and kidneys [19,20]. In the kidneys, they are mainly located in the S2 and S3 segments of the renal proximal tubules and reabsorb most of the filtrated urinary glucose (Table 1) [19,20,21]. SGLT2is inhibit this process and induce glycosuria completely independently of insulin [22]. Since discovering phlorizin as the first SGLT2 inhibitor, several forms of these drugs have been introduced that have all reduced the blood glucose almost to the level of the capacity of nephrons for glucose reabsorption [23,24].

Beyond their potent glucose-lowering effects, they have other pharmacological effects such as glycogenesis suppression, improvement in the insulin sensitivity of peripheral tissues, enhancing the glucagon releasing response, and the induction of insulin secretion from pancreatic β cells [25,26,27,28]. Canagliflozin, dapagliflozin, and empagliflozin are well-known SGLT2 inhibitors with these effects [29,30,31]. However, they also have some adverse effects (e.g., dehydration, dizziness, hypotension, genital infections, and fainting) [29].

## 3. Lipids

**Figure 2:**
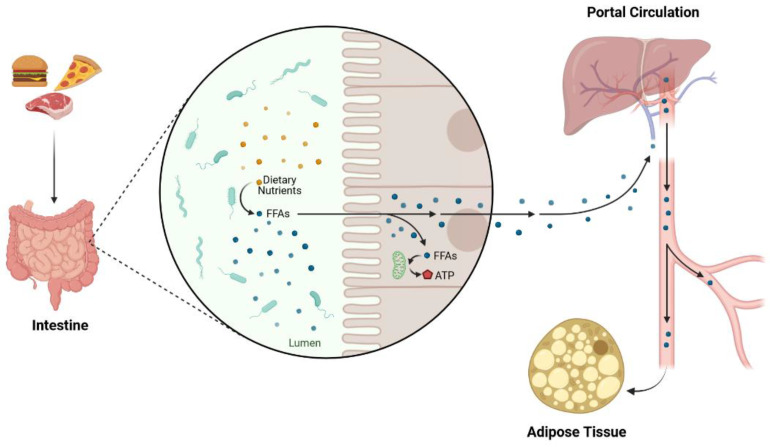
Summary of the nutrients’ absorption, lipid synthesis, and storage in adipose tissue.

Lipids are macromolecules containing hydrocarbons that are only soluble in nonpolar solvents [32]. They are present in the organism because of ingested foods and nutrient absorption in the intestine, but also because of endogenous synthesis (Figure 2) [33]. Although lipids and their combination with apolipoproteins in serum—lipoproteins—have different classifications, triglycerides (TG), cholesterol (Chol), phospholipids (PL), and lipoproteins such as HDL [high density lipoprotein], LDL [low density lipoproteins] and VLDL [very low density lipoproteins]) are recognized as the main forms of lipids and lipoproteins [34]. Sphingolipids, glycolipids, prostaglandins, and free fatty acids (FFAs) (which are simple long-chain hydrocarbon organic acids having a common formula—C_N_H_N_COOH) are also considered to be lipids [34]. While TGs and PLs are made of FFAS, Chol has no complete FFA (except its nucleus which is made of FFAs) in its structure, but it has many of the biochemical characteristics of the lipids [34].

They have important physiological, structural, and functional roles such as energy storage and signaling functions [34]. They are incorporated in the structure of the eukaryotic cell membranes (i.e., lipid bilayers) by making a double layer surrounding the cell cytoplasm [34]. They are also the basis for steroid hormone synthesis such as vitamin D3, prostaglandins, sex hormones, and adrenal steroids (glucocorticoids and mineralocorticoids) [34]. Lipids are the most important form of stored energy in tissues, particularly in adipose tissue (AT), and are an important source of required energy by the body [35]. AT is a mass of aggregated adipocytes (mainly storing TGs and PLs) that was earlier considered as an inert tissue storing energy, but later findings have demonstrated that it has additional important functions in synthesizing and releasing adipokines (adipocytokines) and adiponectin [36,37]. These adipocyte-derived cytokines have significant effects on metabolic pathways and lipid and glucose homeostasis [35]. Therefore, AT is now recognized as an endocrine organ with very interesting and important metabolic potentials [38,39].

## 4. Lipid Metabolism in Diabetes with Focus on Adverse Effects of Diabetes on Lipoproteins

Considering the biological importance of lipids, their normal metabolism is essential for body homeostasis and energy balance [33]. Lipid metabolism is involved in different processes such as lipoprotein synthesis, change and degradation, absorption, and polymerization [40]. These processes are strictly controlled and have a very delicate dynamic equilibrium [40]. This equilibrium determines the total body fat mass. While some lipids are metabolized, oxidized, and used for physiological needs, others are replaced, synthesized, and stored [5,40]. Lipid metabolism is controlled by many endogenous and exogenous factors such as hormones (e.g., thyroid and growth hormones), adipokines and adiponectines, adrenal steroids, sex steroids, and neuronal stimuli [5]. Moreover, other stimuli such as physical activity, feeding style, and social and psychological stressors are able to modify the lipid metabolism [41].

**Figure 3:**
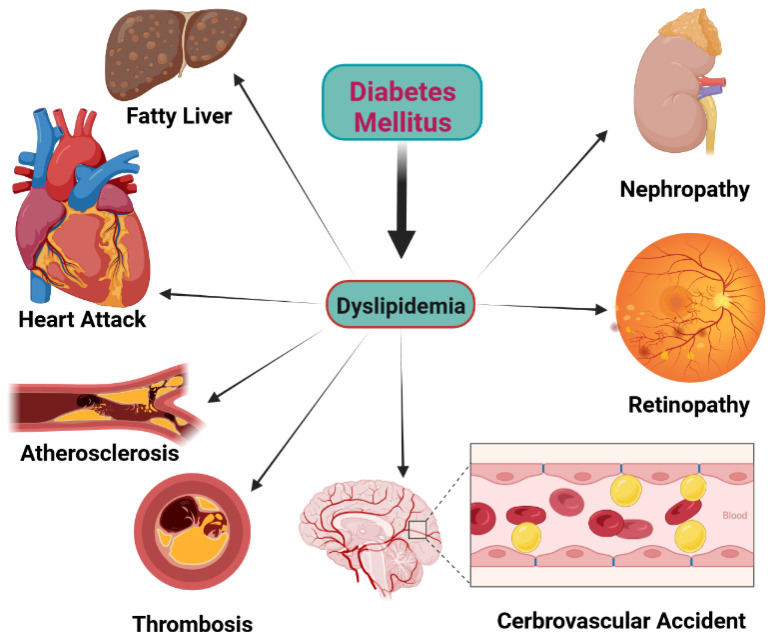
The main forms of dyslipidemia-induced diabetic complications.

Lipid homeostasis is involved in the normal structure and function of most physiologic systems (e.g., cardiovascular, renal, retinal, and neuronal network) [42,43,44]. While lipids are necessary substrates for energy production and signaling as well as structural functions, pathological lipid metabolism or dyslipidemia is directly associated with many life-threatening disorders such as atherosclerosis and its complications (acute myocardial infarction, coronary heart disease, ischemic stroke), cancer, nephropathy, liver damage, and thrombosis [45,46,47,48]. Chronic hypercholesterolemia, which is closely associated with DM, causes primary atherogenesis and myocardial infarction and ischemic strokes [49] as well as renal [50] and retinal diseases (Figure 3) [51,52]. In the diabetic milieu, due to the changes in normal physiological metabolism, the conditions are more suitable for the development of dyslipidemia, and thereby, many diabetic patients have different degrees of dyslipidemia [46,53,54]. Dyslipidemia participates in diabetic complication genesis by several pathologic pathways (e.g., atheroma, plaque formation, and prevention of adequate oxygenation) [54,55,56]. It can produce harmful by-products (e.g., reactive oxygen species (ROS)) and release them [57]. Moreover, it makes a vicious cycle with insulin resistance in which dyslipidemia and insulin resistance stimulate each other [39]. Thus, many diabetic patients have to take lipid-lowering drugs in addition to antidiabetic drugs in order to improve lipid homeostasis and prevent the dangerous consequences of dyslipidemia [3].

## 5. SGLT2 Inhibitors and Lipid Metabolism

Recent evidence has indicated that SGLT2 inhibitors have modulatory effects on lipid metabolism [16]. However, the mechanisms by which they achieve these are not clear thus far. In the following sections, these effects will be discussed.

### 5.1. Biogenesis of Lipids

Lipogenesis and lipolysis are two key determinants of lipid homeostasis that determine the amount of total body fat mass and are highly controlled metabolic processes [58,59]. Lipogenesis is a metabolic process in which FFA and TG are synthesized from different substrates (e.g., carbohydrates, acetyl-coenzyme A (CoA), and glycerol) in the mitochondria and smooth endoplasmic reticulum [60]. This process primarily occurs in the liver and, to a certain extent, in adipose tissue. However, in individuals with low physical activity and high caloric diet input, adipose tissue is the major site of lipogenesis [61]. It also occurs to some extent in other tissues (e.g., kidneys, brain, lungs, and intestine) [62]. On the other hand, lipolysis is the other important metabolic process of lipid metabolism during which TGs break down into FFAs and glycerol by hydrolysis, mainly at the surface of the cytosolic lipid droplets of adipocytes [63]. This process mainly occurs in white adipose tissue during fasting to supply the necessary metabolic substrates and energy [63,64]. There is a delicate equilibrium between lipolysis and lipogenesis. However, in a pathological milieu such as diabetes, this balance is disturbed and dyslipidemia develops [3]. Pathologic states of lipolysis and lipogenesis are closely associated with metabolic disorders such as obesity, DM, non-alcoholic fatty liver disease (NAFLD), insulin resistance, and atherosclerosis (Figure 3) [47,48,65,66].

**Figure 4:**
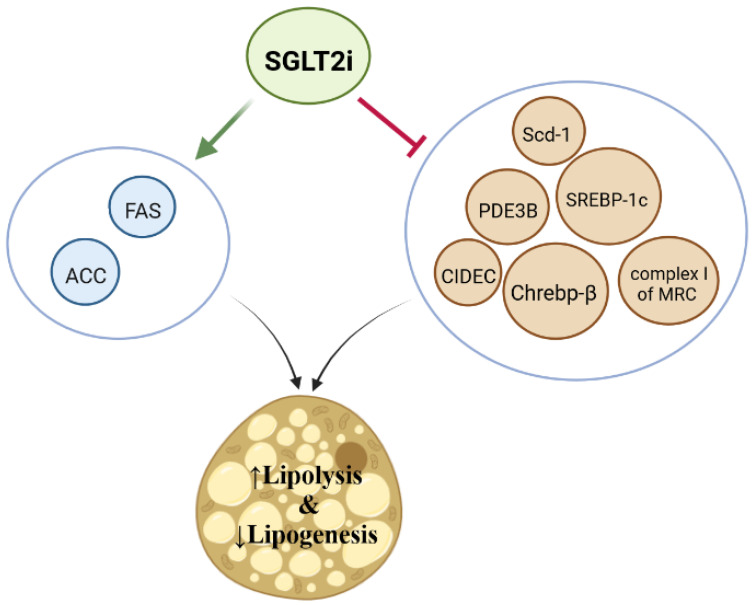
Possible mechanisms involved in SGLT2i dependent lipid biogenesis. SGLT2 inhibition reduces lipogenesis and induces lipolysis upregulating ACC (acetyl-CoA carboxylase) and FAS (fatty acid synthase) and downregulating Scd-1 (Stearoyl-CoA desaturase-1), PDE38 (Phosphodiesterase 3B), SREBP-1c (sterol response element-binding protein 1c), CIDEC (Cell death inducing-DFFA like effector C), Chrebp-β (Carbohydrate response element-binding protein-beta), and the activity of complex 1 of MRC.

SGLT2 inhibitors have significant effects on lipolysis and lipogenesis [27]. Jojima et al. reported that empagliflozin decreased de novo lipogenesis in diabetic mice [67]. They found that 3 weeks of empagliflozin therapy inhibited fatty liver development by the downregulation of FAS (fatty acid synthase) and ACC (acetyl-CoA carboxylase) and decreased lipogenesis in the hepatocytes of diabetic mice [67]. Lauritsen et al. recently found that 4 weeks of empagliflozin therapy increased circulating FFA and induced lipolysis in patients with T2 (type 2) DM [68]. They suggested that empagliflozin had this effect most likely because of the downregulation of CIDEC (Cell Death Inducing DFFA Like Effector C—a regulator of adipocyte lipid metabolism) and PDE3B (Phosphodiesterase 3B—a key regulator of lipolysis and energy homeostasis) [68]. Moreover, Osataphan et al. reported that SGLT2 inhibition with canagliflozin improved in nondiabetic mouse lipogenesis by decreasing the expression levels of genes involved in de novo lipogenesis such as Chrebp-β (Carbohydrate response element-binding protein-β—an important transcription factor in de novo lipogenesis), ACC, FAS, and Scd-1 (Stearoyl-CoA desaturase-1—a key enzyme in FFA metabolism) [69]. They demonstrated that canagliflozin induced lipolysis in white adipose tissue probably via FGF21 (fibroblast growth factor 21)-dependent mechanisms and lipolysis reprogramming at the transcriptional level [69]. Day et al. reported that canagliflozin suppressed hepatic lipid synthesis and the expression of ATP-citrate lyase, acetyl-CoA carboxylase, and SREBP-1c (sterol response element-binding protein 1c—a key regulator of FFA metabolism) and sterol response [70]. In another study, canagliflozin activated AMPK (adenosine monophosphate-activated protein kinase) by inhibiting complex I of MRC (mitochondrial respiratory chain), which in turn phosphorylated and activated the ACC and reduced lipogenesis in the liver tissue of mice [71]. Taken together, SGLT2 inhibitors can stimulate lipolysis and inhibit lipogenesis by several cellular pathways (Figure 4).

### 5.2. Cholesterol Homeostasis

Cholesterol is an important lipid molecule with important roles in steroid and hormone synthesis and the structure of the cell membrane [72]. Its circulating level is highly associated with the risk of atheroma plaque formation and atherosclerotic cardio-vascular diseases [73]. Therefore, normalizing its level and keeping its normal homeostasis is essential in preventing atherogenesis [36]. There are different reports about the effects of SGLT2i therapy on cholesterol metabolism and while some have suggested beneficial effects [69], the others reported adverse [74] or no significant effects [75].

There is evidence suggesting that SGLT2i therapy with canagliflozin or dapagliflozin can reduce the cholesterol levels [69]. For example, Osataphan et al. demonstrated that canagliflozin reduced circulating cholesterol levels in mice through the inhibition of genes involved in its uptake (PCSK9) and synthesis (Hmgcr, Lss, and Hmgcs1) [69]. Gürkan et al. reported that 6 months of dapagliflozin therapy decreased the serum levels of LDL-cholesterol and TGs in T2DM patients [76]. Moreover, in another clinical study, 6 months of SGLT2i therapy with luseogliflozin decreased the LDL-cholesterol and increased the HDL-cholesterol levels in T2DM patients [77].

However, some evidence suggests that SGLT2 inhibition increases the LDL-cholesterol and total cholesterol levels [74]. For example, Basu et al. demonstrated that canagliflozin increased the circulating TG, LDL-cholesterol, and total cholesterol levels in diabetic mice, probably due to increased LPL (lipoprotein lipase) activity, decreased postprandial lipemia, and faster clearance of VLDL from circulation [74]. They concluded that SGLT2 inhibition was correlated with increased lipolysis (LPL activity) and increased substrate for cholesterol synthesis [74]. A systematic review and meta-analysis of RCTs (randomized controlled trials) showed that SGLT2 inhibitors increased the total cholesterol, LDL-cholesterol, and HDL cholesterol, and decreased the TG levels in patients with T2DM [78]. Another clinical trial demonstrated that after 12 weeks of canagliflozin treatment, HDL-cholesterol levels were increased, thus providing cardiovascular benefits in patients with T2DM [79]. This benefit occurred at high-dose therapy [80]. Shi et al. reported that high dose of SGLT2, inhibitors of canagliflozin and dapagliflozin, increased HDL-cholesterol and decreased TG levels in patients with diabetes [80]. Moreover, Cha et al. found that SGLT2i therapy for 24 weeks was related to a significant increase in HDL-C and LDL-C in patients with T2DM [81].

Nevertheless, some evidence indicated no significant improvement in the cholesterol profile after SGLT2i therapy [75]. Fadini et al. demonstrated, in a clinical trial on patients with T2DM, that 12 weeks of dapagliflozin therapy had no significant effect on HDL-cholesterol, although it decreased the cholesterol efflux capacity from macrophages [75]. Bosch et al. performed a clinical trial on patients with T2DM and found that empagliflozin had no significant effect on the total cholesterol, HDL-cholesterol, and LDL-cholesterol levels [82]. Another more recent study also showed that treatment with luseogliflozin and voglibose had no significant effect on the cholesterol profile in T2DM patients [77]. It showed that luseogliflozin had no significant effect on malondialdehyde, LDL particles, and small dense LDL particle cholesterol [77]. It seems that SGLT2 inhibitors have different effects on cholesterol metabolism and may increase, decrease, or not have any effect on its level, and the mechanisms by which any of these actions are performed are still unknown.

### 5.3. Fatty Acid Uptake and Utilization (β-Oxidation)

FFA β-oxidation is a poly-phasic enzymatic process by which FFAs are broken down into their components to provide the necessary energy [83]. It mainly occurs in mitochondria (as well as in peroxisomes) to produce acetyl-coA [83]. Acetyl-coA is then converted into NADH (nicotinamide adenine dinucleotide); the main substrate for ATP (adenosine triphosphate) production; which in turn enters the Krebs (tricarboxilic acid [TCA]) cycle and mitochondrial electron transport chain to generate the required energy [83]. Since FFAs are burned out during this catabolic process, FFA β-oxidation has a significant role in lipid homeostasis and energy balance [83,84]. It is regulated at two main levels—the transcriptional and post-transcriptional (or allosteric) levels [83]. While key regulatory proteins such as PPARs (peroxisome proliferator-activated receptors), SREBP1, and PGC-1α are responsible for transcriptional control, allosteric control is performed by by-products that have their effects on enzymes participating in metabolism through negative or positive feedback [83,85].

There is evidence to suggest that SGLT2 inhibitors modify FFA β-oxidation [86]. A recent study on rats demonstrated that 4 weeks of dapagliflozin therapy induced FFA oxidation instead of storing them in liver tissue [86]. These authors found that this effect was associated with increased clearance and flux of FFAs by β-oxidation and higher acetyl-CoA concentrations [86]. In a clinical study, empagliflozin had no significant effect on FFA uptake in myocardial tissue, but it reduced myocardial glucose uptake in patients with T2DM [87]. More strong evidence provided recently by Herring et al. [88] indicated that dapagliflozin increased FFA β-oxidation toward more ketone body production in people with T2DM. Kroon et al. [89] demonstrated that 4 weeks of dapagliflozin therapy was associated with increased circulating levels of FFAs and enhanced hepatic FFA oxidation and ketone body formation in rats. Although some studies have reported no significant effects on lipid metabolism for these drugs [31], or have demonstrated that they may reduce FFA oxidation in high-fat diets (to prevent subsequent injury due to high lipid utilization) [90], most studies have suggested that SGLT2 inhibition prevents FFA from accumulating in adipose tissue and directs them toward oxidation and utilization to provide the needed energy in the shortage of available carbohydrates (due to glycosuria) [68]. However, the effects of these drugs on enzymes involved in FFA oxidation (e.g., acyl-CoA dehydrogenase, enoyl-CoA hydratase, hydroxyacyl-CoA dehydrogenase, and ketoacyl-CoA thiolase) [91] have not been thoroughly investigated, which need further studies.

### 5.4. Lipid Peroxidation

The oxidative degradation of lipids or lipid peroxidation is a pathological cellular event in which free radical species steal electrons from the lipids of cellular membranes and produce toxic metabolic by-products (e.g., F2-isoprostanes, malondialdehyde (MDA), thiobarbituric acid reactive substances (TBARS) and 4-hydroxynonenal (4HNE)) [92,93]. Lipid hydroxides can in turn induce oxidative injury and produce oxidative (or nitrosative) stress [94]. They can also bind to specific parts of DNA and have mutagenic effects by forming DNA adducts and different biomarkers such as 8-oxo-2′-deoxyguanosine (8oxodG), especially in a weakened antioxidant defense system, which is in the diabetic milieu [95,96]. Therefore, the inhibition or reduction in lipid peroxidation by antioxidant agents in the biological milieu is an important target to prevent subsequent cellular injuries [95].

There is only limited evidence about the role of SGLT2 inhibitors on lipid peroxidation [19,97]. However, since they can have antioxidative effects, it may be concluded that they are able to reduce lipid-peroxidation [97]. In a recent study, dapagliflozin decreased DM-dependent oxidative damage in the cardiomyocytes of mice [97]. These authors showed that dapagliflozin downregulated NADPH oxidase (Nox; a potent oxidative enzyme)-dependent ROS production and MDA levels in the cardiac tissues of these animals [97]. In another study, SGLT2 inhibition reduced lipid peroxide metabolites such as MDA and 4HNE in the kidneys [98]. Kimura et al. showed that canagliflozin developed these effects through the reduction in the Nox expression in the renal tissues of diabetic rats [98]. Similarly, Oshima et al. found that empagliflozin prevented oxidative stress and lipid peroxidation dependent cellular death through the increase in myocardial levels of Sirt3 (Sirtuin 3—an important protein for maintaining mitochondrial integrity and function) and SOD (superoxide dismutase) in the myocardium of diabetic rats [99]. They reported that empagliflozin reduced the MDA and 4HNE levels in the treated animals [99]. Dapagliflozin has also shown the same effects in another study [100]. It has shown cardio-protective benefits through a reduction in oxidative injury and lipid peroxidation by-products in cardiomyocytes and human breast cancer cells [100]. This evidence suggests that SGLT2 inhibitors can reduce lipid peroxidation due to their antioxidative effects. It seems that these drugs induce antioxidative defense systems and reduce the expression of oxidative agents, thus decreasing the oxidative injury and lipid peroxidation.

### 5.5. Lipid Absorption/Transport

The amount of dietary lipid absorption and/or transport has an impact on whole body fat mass and risk of hyperlipidemia [101]. Plasma lipid levels are the key determinant of hepatic lipid biosynthesis and circulating levels of HDL-cholesterol, LDL-cholesterol, and VLDL [102,103]. Lower levels of dietary lipid uptake are associated with lower circulating TG and LDL-cholesterol levels as well as lower risks of adiposity, dyslipidemia, and atheroma plague formation. Using lipid uptake inhibitors in high-risk patients might be an efficient method to prevent hyperlipidemia and subsequent atherosclerotic diseases [104].

There is limited evidence suggesting that SGLT2 inhibitors could modify lipid transport [68]. Lauritsen et al. recently found that SGLT2 inhibition with empagliflozin for 4 weeks reduced the GLUT4 gene and protein expression in abdominal adipose tissue, which could indicate a rebalancing of substrate utilization from glucose oxidation and lipid storage to reduce the glycerol formation in patients with T2DM [68]. They suggested that empagliflozin therapy increased lipid mobilization and reduced the accumulation of lipids in abdominal adipose tissue [68]. Similarly, Wallenius et al. recently demonstrated that 4 weeks of SGLT2 inhibition with dapagliflozin increased FFA mobilization and transport from adipose tissue in obese rats [86]. SGLT2 inhibitors reduced GLUT4 expression in adipocytes [68], and can thereby mobilize ingested lipids toward lipolysis and β-oxidation [86]. Currently, we have no evidence concerning the role of these drugs on lipid absorption, and therefore, this question requires further studies to explain this.

## 6. Conclusions

SGLT2 inhibitors are a relatively novel class of antidiabetic drugs, which provide potent glucose-lowering effects through the induction of glycosuria. Recent evidence has suggested that these drugs could provide additional benefits apart from their glucose-lowering effects by normalizing lipid metabolism, and therefore, they are able to prevent diabetes-induced dyslipidemia and diseases caused by dyslipidemia (Table 2). This review showed that SGLT2 inhibitors modulated lipid metabolism by at least five cellular pathways: lipid biogenesis (lipogenesis and lipolysis), lipid peroxidation, lipid transport, cholesterol biosynthesis, and fatty acid β-oxidation. However, the adverse effects concerning their effects on cholesterol and the benefits of these drugs were also described. In this article, strong clinical evidence based upon experimental and clinical data was presented. Therefore, it could be concluded that SGLT2 inhibitors might be promising antidiabetes drugs, especially in patients with dyslipidemia. Nevertheless, further studies, especially long-term clinical trials, are still required to fully understand the lipid-lowering and lipid-modifying effects of these drugs.

## Figures and Tables

**Table 1:** The main characteristics of the SGLT2 protein and its inhibitors.

Protein	Location (Kidney)	Involvement in Glucose Reabsorption	Affinity for Glucose	Capacity	Na^+^/Glucose Transport Ratio	Main Inhibitors
SGLT2	S_1_ and S_2_ segment of proximal tubules	~90%	Low	High	1:1	Dapagliflozin, Canagliflozin, Luseogliflozin, Sotagliflozin

**Table 2:** The effects of SGLT2 inhibitors on lipid metabolism.

Lipid Metabolism	Used SGLT2 Inhibitors	Effects of SGLT2 Inhibition	Refs.	Clinical Evidence
Lipogenesis and Lipolysis	Empagliflozin, Canagliflozin	Induces lipolysis and inhibits lipogenesis	[67,69]	[68]
Lipid Peroxidation	Empagliflozin, Dapagliflozin, Canagliflozin	Reduces lipid peroxidation and oxidative damages	[97,98,99,100]	[77]
Fatty Acid β-Oxidation	Dapagliflozin	Induces and promotes FFAs oxidation/utilization	[86]	[87,88]
Cholesterol Homeostasis	Canagliflozin	Increases cholesterol level	[74]	[81]
	Canagliflozin	Decreases circulating cholesterol levels	[69]	[76]
	Luseogliflozin, Dapagliflozin, Empagliflozin	No significant effects	-	[75,77,82]
Lipid Absorption/Transport	Dapagliflozin	Mobilizes ingested/stored lipids and diverts them from adipose tissues	[86]	[68]

## Data Availability

Not applicable.
